# Our Friend the “Peninsular Journal”

**Published:** 1857-04

**Authors:** 


*Our Friend the "Peninsular Journal."*—The "Peninsular," in replying to our paragraph in the February number, answers that it has no wishes concerning the correspondence alluded to by us, and adds that that correspondence was not the sin laid at the door of the Buffalo Medical College. This answer renders it unnecessary to consider that subject further, except that we wish to keep its existence in remembrance, and may possibly conclude to give it a general circulation at some fitting time, through the medium of the criminal courts.

What the Peninsular did mean is this—in its own language:

"Not long since we met with one of the few students who were at the Buffalo Medical School during the session before the present, *and* who informed us that one of the members of that Faculty, in a speech to the class, told them some marvelous things about the Medical Department of the University of Michigan—speaking most disparagingly of the school and its faculty, saying that he spoke from personal knowledge of some of the men, &c. The individual member of the Buffalo faculty, particularly spoken of as making these offensive charges, we have not the privilege of personally knowing, but he will understand who and what is meant. We have the name, and have confidence in the integrity of our informant. Does the Buffalo Journal wish us to be more explicit? It is very natural for men to be jealous of a successful rival, and under the circumstances we can forgive a good deal. But even the small number of students at Buffalo, as compared with those in the Medical Department of the University of Michigan, will not justify, or render honorable, the language we are credibly informed was used."

Verily this is a serious matter. Here is our answer thereto:

1st. Members of the Buffalo faculty are not in the habit of making speeches to their classes, but usually confine themselves to the subject of the day. The University of Michigan is a bit of morbid pathology—a sort of *noli me tangere*—which is not included in the curriculum of the school.

2d. The individual member who made this speech—if any was made—doubtless "spoke from personal knowledge of some of the men, &c." We are ready to admit that count, and as we have none but men of the strictest veracity in the faculty of Buffalo, he doubtless spoke the truth so far as in him lay—probably not the whole truth, however.

3d. We have not the "privilege of personally knowing" who made the speech in question, never having heard of it before, but we agree with the

*Peninsular*, that the sinner, whoever he is, "will understand who and what is meant." With the *Peninsular*, also, we have confidence in the integrity of its informant. At any rate we are sure he heard no compliments.

4th. In reply to the question whether we wish the Peninsular to be more explicit, we must transpose its own language—thus. "The *Buffalo Journal* has no particular interest in the matter, and has no desires respecting the publication, pro or con, and if it had, has no power to consent or refuse."

And now for the remaining charge—the only one which is essential—that of jealousy on the part of the Buffalo School of her successful rival, the Ann Arbor institution.

We can only answer for ourselves and shall speak our opinions with a freedom which would be unjustifiable were it not in response to repeated taunts upon the "small number of students at Buffalo." Is there any special matter for boasting in the success of the medical department at Ann Arbor? What has it accomplished of which we should be jealous? It has thrown open its doors to every one gratuitously—its charities not confined to its own state but offered to the young men of every other and of Canada—by constant and systematic puffing, by unlimited distribution of its boasting circulars, by loud assertion of the important fact that their lecture fees are nothing, and their graduation fee three dollars, added to alluring accounts of the cheapness of living at Ann Arbor, they have drawn together a hundred and fifty students, and this they call success.

Delighted with accomplishing so much, they have gone further, and secure in their position as pensioners of a state government, they have been mean enough to taunt self-supporting schools with their poverty and small classes. Let our Michigan competitor place itself on an equality with the humblest of the chartered schools; let it place its tuition fees at thirty dollars, as we hear the Geneva school has done, and it will then be able to measure its success by some decent rule of comparison. As it is, the Buffalo school, located in a city, with of course city expenses, and a higher tuition fee than any of its neighbors, has only one-third the number of students that congregate at Ann Arbor. We plead guilty to this without a blush.

We cannot of course institute a comparison of the scholarship or advantages of the two schools. The *Peninsular* has implied such a comparison in its insulting allusions, but we prefer leaving that subject without argument, to the profession at large as a jury. But we are justified in saying here, that the Ann Arbor school has failed to realize the expectations formed from the favorable circumstances of its organization, that placed in a situation which enabled it to raise the standard of graduation, and send out its students to the world with a guarantee that, however great the disadvantages of their country location, they had redeemed them by rigid rules of qualification for the doctorate, they have taken no step forward in any comprehensive reform, and the reputation of their diploma is as low as that of any other in the land, without exception.

